# T2 abnormalities in patients with hypertrophic cardiomyopathy characterized by cardiovascular magnetic resonance imaging- an indicator of myocardial injury as assessed by the high sensitive cardiac troponin T assay

**DOI:** 10.1186/1532-429X-14-S1-O100

**Published:** 2012-02-01

**Authors:** Stephanie Lehrke, Dirk Lossnitzer, Diana Viertler, Evangelos Giannitsis, Henning Steen

**Affiliations:** 1 Diagnostic and Interventional Radiology and Neuroradiology, Ev.- Lutherische Diakonissenanstalt Flensburg, Flensburg, Germany; 2 Cardiology, University of Heidelberg, Heidelberg, Germany

## Summary

To test the hypothesis that myocardial hyper- intensities on T2 weighted imaging in patients with hypertrophic cardiomyopathy (HCM) reflect ongoing myocardial injury, the cardiac- specific biomarker troponin T was measured by a new highly sensitive assay (hs-cTnT). In 52 patients with HCM, the presence and extent of myocardial T2 abnormalities were associated with hs-cTnT levels. The number of LV segments with T2 hyper-intesity was the strongest independent predictor of hs-cTnT levels. This novel finding suggests that T2 imaging may aid in the identification of HCM patients with active and potentially progressive disease.

## Background

In a sub-group of patients with hypertrophic cardiomyopathy (HCM) abnormalities on T2 weighted imaging on cardiovascular magnetic resonance imaging (CMR) have been reported. The significance of this finding is still not clear, although it has been proposed to reflect ongoing myocardial injury. The loss of cardiomyocyte cell integrity leads to the release of troponins into the circulation. Recently, a highly sensitive cardiac troponin T assay (hs-cTnT) was introduced which allows the detection and quantification of very low serum levels of cTnT with a high diagnostic accuracy.

We therefore aimed to test the hypothesis that the presence and extent of myocardium with increased signal intensity on T2-weighted images in patients with HCM is associated with evidence of cardiomyocyte injury as assessed by the hs-cTnT assay.

## Methods

The study cohort consists of 52 patients undergoing late gadolinium enhanced CMR (LGE-CMR) on a 1.5T clinical scanner (Philips Intera Achieva®) for characterization of hypertrophic cardiomyopathy in whom hs-cTnT was measured by the ELECSYS® assay (Roche Diagnostics). Left ventricular (LV) volumes and mass were derived from SSFP images. The presence of hyper-intense areas on T2 images was assessed in the modified LV AHA-16 segment model by two blinded observers. LGE was quantified in percent of LV mass.

## Results

T2 hyper-intensities were present in 31 (60%) patients (T2-pos) with a mean of 2.67± 1.6 LV-segments affected. The presence of T2 hyper-intensity was associated with increased septal wall thickness (12± 4.9 mm vs. 16.9± 3.6 mm, p=0.05) and the presence (100% vs. 44.4%, p < 0.001) and extent of LGE (2.7± 5.9 % vs. 15.8± 14.9 %, p=0.002). T2-pos patients showed markedly increased levels of hs-cTnT (20.2± 16.4 pg/ml vs. 9.8± 7.5 pg/ml, p=0.02). Furthermore, the number of myocardial segments showing a signal hyper-intensity on T2 imaging was correlated to hs-cTnT levels (R=0.52, p=0.02). Remarkably, when entered into a multivariate logistic regression model, the number of T2 segments was the only independent predictor of hs-cTnT levels in addition to renal function (table1).

**Table 1:** Predictors of continuous hs-cTnT levels

	B	95% CI of B	p	r^2^
**Univariate**				

**Age (per year)**	0.26	0.2-0.32	0.001	0.58
**CAD**	26.2	11.1-41.2	0.001	0.54
**Creatinin (per mg/dl)**	15.3	11.6-19.4	< 0.001	0.76
**LV-EF (per %)**	0.21	0.14-0.27	< 0.001	0.67
**LV-T2 (per segment)**	7	5-8.9	< 0.001	0.76
**LGE volume (per %)**	0.84	0.58-1.1	< 0.001	0.7

Multivariate^*^				

**LV-T2 (per segment)**	4.7	1.8-7.5	0.0003	0.87
**creatinin**	12.3	4.8-19.8	0.002	

CAD:coronary artery disease; LV: left ventricular; EF: ejection fraction;LGE: late gadolinium enhancement

^*^ the multivariate model contains all listed univariate variables

## Conclusions

The presence and the extent of myocardial T2 abnormalities in patients with HCM predict serum levels of hs-cTnT and thus seem to reflect ongoing myocardial damage. Whether this finding is associated with an increased risk for future adverse events needs to be examined in further studies.

## Funding

None.

